# Mesmerism at the Westminster Aquarium

**Published:** 1890-06-28

**Authors:** 


### MESMERISM AT THE WESTMINSTER AQUARIUM.

AN American, named Kennedy, is giving illustrations of mesmerism at the Aquarium. We have attended an exhibition, which is now briefly described. Mr. Kennedy was a quarter of an hour late in appearing—much to the dissatisfaction of the audience. He accounted for the delay by complaining of hoarseness, which required much time for periodical medicine. Evidently, therefore, Mr. Kennedy does not believe in mesmerism as a specific for this particular complaint. Mr. Kennedy's first procedure was to ask if three medical men from the audience would ascend the stage for the purpose of seeing "fair play." Whereupon three young gentlemen mounted. But the audience were not satisfied, said the three young gentlemen could only be medical students, and asked what qualifications they possessed. As an answer was not readily afforded, a gentleman ascended, who said he was a medical man, but refused to give name or qualifications. Ultimately another gentleman appeared, who announced his name and qualifications. This seemed satisfactory to the auditory, and the storm was stayed. Then Mr. Kennedy called for volunteers who had no objection to submit themselves to the mesmeric process. More than twenty youths and men responded and seated themselves on chairs, previously placed in a semi-circle round the back of the stage. The operator then told them he was about to try to mesmerise them. They were asked to place their hands on their thighs, and to close their eyes. The operator then passed round, looking at each in turn, and again touching the forehead of each in turn. He then told them they were not to open their eyes, as they were asleep. With the exception of two or three this soon proved to be correct. One after the other, at different intervals, reclined his head on his neighbour's shoulder, the majority ultimately falling to the floor in most uncomfortable attitudes. All this occupied only a few minutes. Then the operator touched one of his "subjects," caused him to assume the erect posture, and brought him to the footlights. Then the operator thrust needles into different parts of the "subject's" body, and passed a needle and long thread through the "subject's" tongue and through his cheek. The medical man on the stage asked permission to operate with the needle, which was immediately accorded. He then ran the needle in, apparently to the hilt, and seemingly dangerously near the brachial artery. Several other subjects underwent a similar test. Then the operator filled the nose and mouth of one of the sleepers with undoubted cayenne pepper. Some pepper was also placed in the eyes. Immediately afterwards the sleeper was roused, and declared he felt no effect from the pepper, but his eyes were certainly bloodshot and watering. One of the sleepers was told his face was dirty, and to go and wash it in "that basin of water," which was in reality a basin of flour. The subject washed himself in flour, with apparent enjoyment. Another youth was told his next-door neighbour was his sweetheart, whereupon he commenced to embrace. A man was told he had a fly on his nose, but his hands being in his pockets, he could not brush it off; but he made the most hideous grimaces under the hallucination of an itching nose. Four gentlemen were told to consider themselves eminent singers, to announce themselves to the audience, and to sing. This they did with the greatest assurance, although one only could sing at all, and he sang the music-hall ditty, "There is no room for me," much to the amusement of the company. While the subjects were singing Mr. Kennedy passed needles and threads through their ears, and through the skin of their cheeks, without interrupting the singing. He also checked the song suddenly, made the subject do something else, and resume the song exactly where left off. Then Mr. Kennedy compounded a vile decoction of oil and kerosine and cayenne pepper which was poured into tumblers. This was tasted by the medical man on the stage, and pronounced "beastly." But two "subjects" being told it was whiskey of fine blend, drank it off with seeming enjoyment, asked for more, and had more! The operator stated they would feel no ill effects from this emetic cathartic dose! Mr. Kennedy then produced a walking stick which half-a-dozen subjects followed about, endeavouring to obtain a bite of it, as they were told it was sugar-candy. A laughing performance followed, the whole of the subjects being convulsed with laughter, in which the audience heartily joined. Two subjects were then rendered cataleptic, and placed so as to rest with their heads and their heels on the backs of two chairs. Mr. Kennedy sat upon them, and although a good fifteen stone (apparently) failed to make any impression. But here it was observed that Mr. Kennedy's hands rested near the head and feet of the cataleptic subject, thereby taking off considerable weight from the centre of the body. The whole of the mesmerized were then told their shoes were filled with molten lead, whereupon they all exhibited the most intense characteristics of pain, hastening to take their boots off. This concluded the entertainment.

Now the general impression of the audience was sceptical, although most admitted "there was something in it." Of the reality of the sleep there could be no doubt, for the pupils of the eyes of the subjects were dilated and insensible to light. Insensibility of the iris cannot be assumed at will. Then people taken promiscuously from a large audience, and taken also unawares, could not endure without flinching, the severe test of needles and long threads being thrust through their tongues and skins. Next it would seem perfectly impossible for anyone in the normal condition to drink the nauseous compound prescribed above without vomiting. There could scarcely be a collusion of twenty persons with Mr. Kennedy. And the play of the features and conduct of the subjects in accordance with what they were told by the mesmeriser were too characteristic to be attained without long education and practice. The names and addresses of the "subjects" were freely given.

We have yet much to learn about mesmerism, electro-biology, animal magnetism, hypnotism, or whatever name the so-called science may be termed. It had a place among the sacred mysteries of the ancient Greeks. And there is much reason to believe it was practised in the middle ages by the professors of the "black art." Mesmer did not initiate the doctrine, but brought into prominence what had been lying dormant. In a former article we referred to the enquiry made by Dr. Braid some for[illegible] years ago, and to Esdaile's operations on the mesmerised. We have also several times referred to Dr. Tuckey's book on Hypnotism, or treatment of disease by sleep and suggestion. And we have mentioned the operations recently performed at Leeds on mesmerised subjects, in the presence of a large number of medical men.

It is a curious fact that an idiot cannot be mesmerised. Neither are all persons susceptible. Mr. Kennedy himself only professes to be able to mesmerise a certain *percentage*. And very few persons are able to mesmerise; therefore, the science, or whatever it may be termed, will, perhaps, never become of any great practical utility. Mr. Kennedy also confesses that although mesmerism has its fascinations it has its dangers. We have heard of amateurs not being able to de-mesmerise their subjects! Two Paris physicians asserted that by hypnotism "the spirit is so far disengaged from the body, that it would be impossible for it to re-enter without the effort of the mesmeriser's will." We are strongly of opinion, that it is a matter which should be taken up authoritatively, so that the dry bones of fact may be separated from the drapery of invention, with which charlatans have long surrounded mesmerism.

